# Supplementary material for: The Association between Vitamin D Status and Autism Spectrum Disorder (ASD): A Systematic Review and Meta-Analysis
Source: Nutrients. 2020 Dec 29;13(1):86. doi: 10.3390/nu13010086 (PMC7824115; doi:10.3390/nu13010086)
Supplement: Supplementary file 1 [file nutrients-13-00086-s001.zip › SUPPL/Suppl Figures.pptx]

## Slide 1
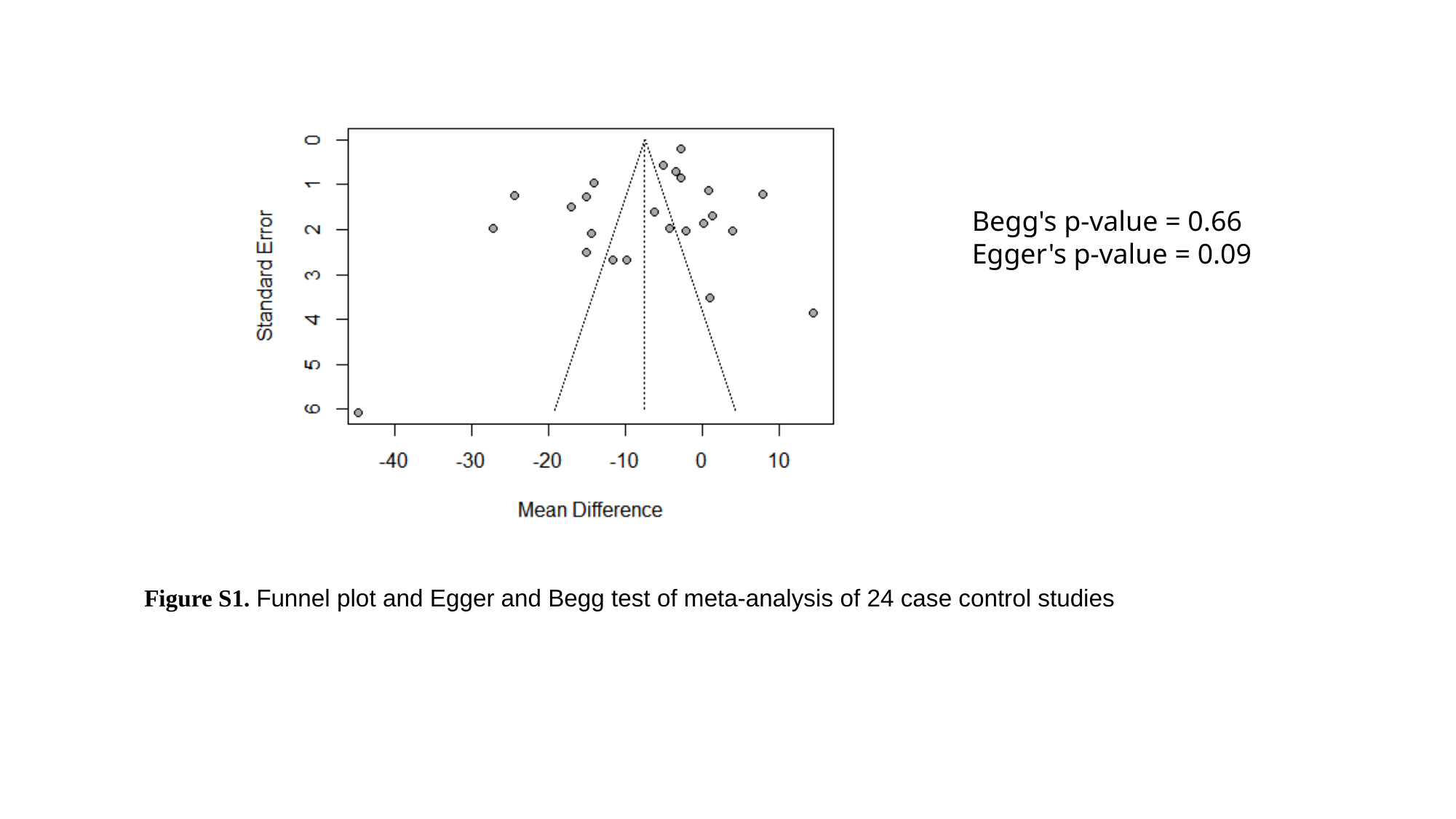

Begg's p-value = 0.66
Egger's p-value = 0.09
Figure S1. Funnel plot and Egger and Begg test of meta-analysis of 24 case control studies

## Slide 2
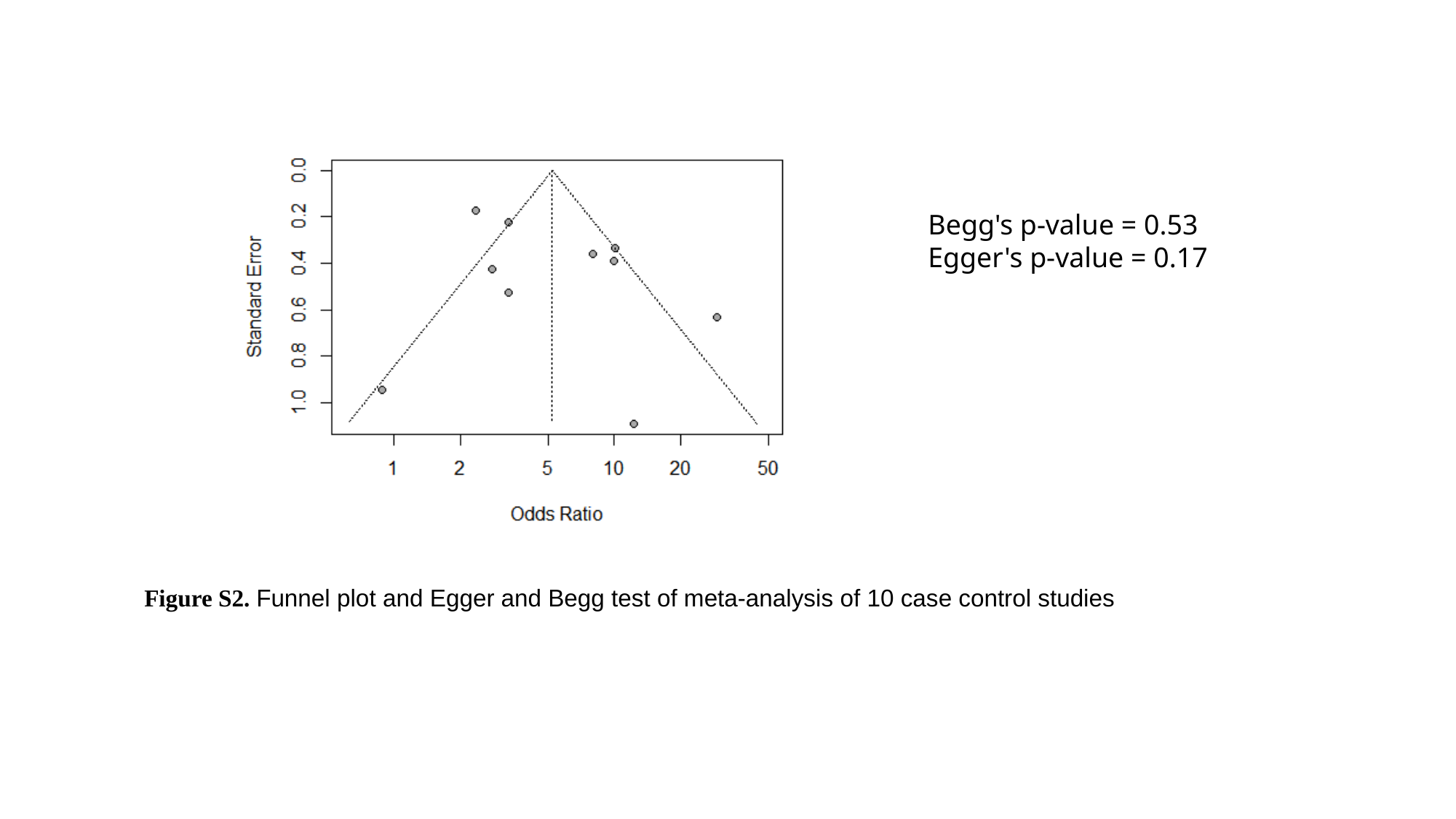

Begg's p-value = 0.53
Egger's p-value = 0.17
Figure S2. Funnel plot and Egger and Begg test of meta-analysis of 10 case control studies

## Slide 3
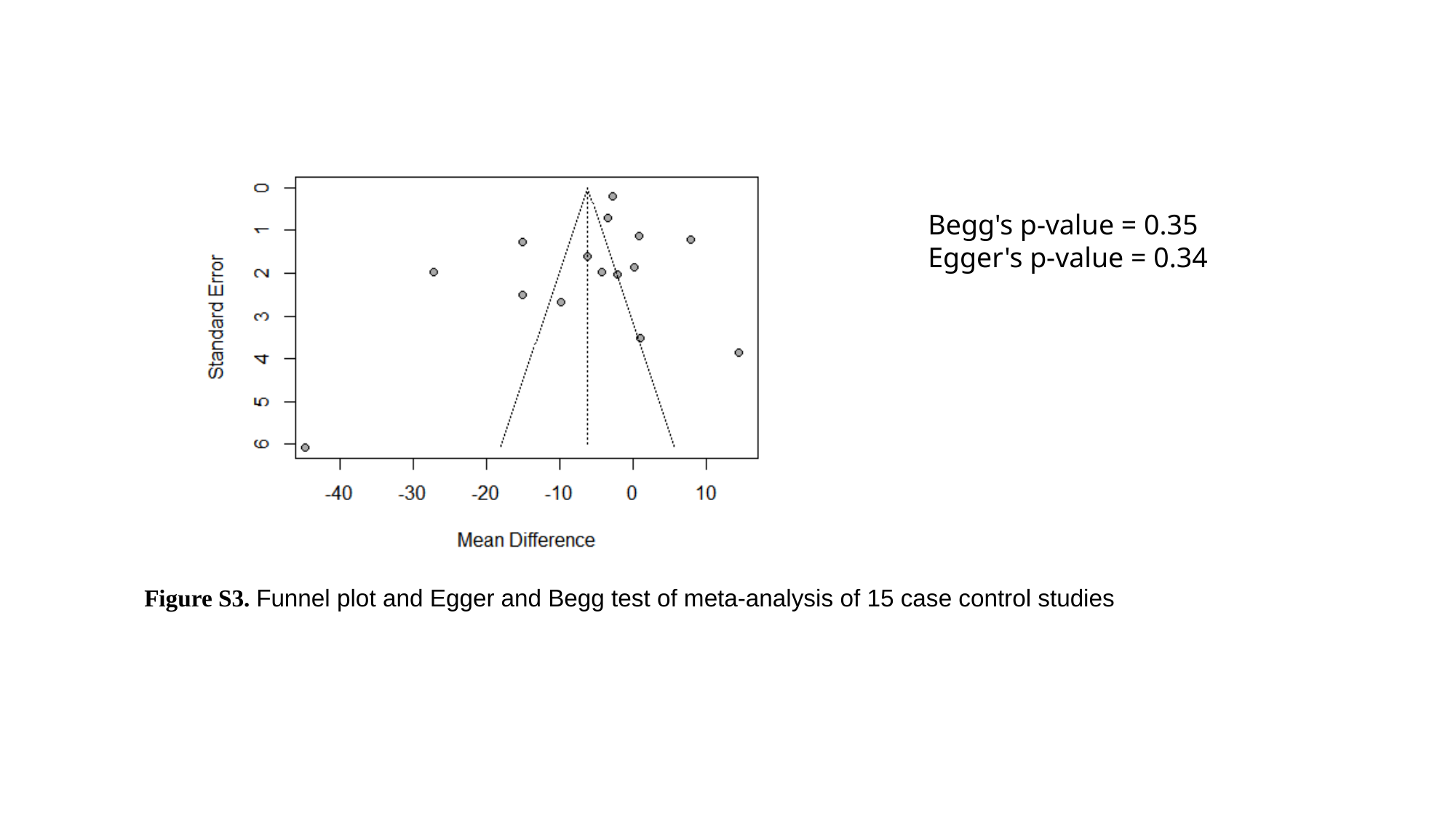

Begg's p-value = 0.35
Egger's p-value = 0.34
Figure S3. Funnel plot and Egger and Begg test of meta-analysis of 15 case control studies

## Slide 4
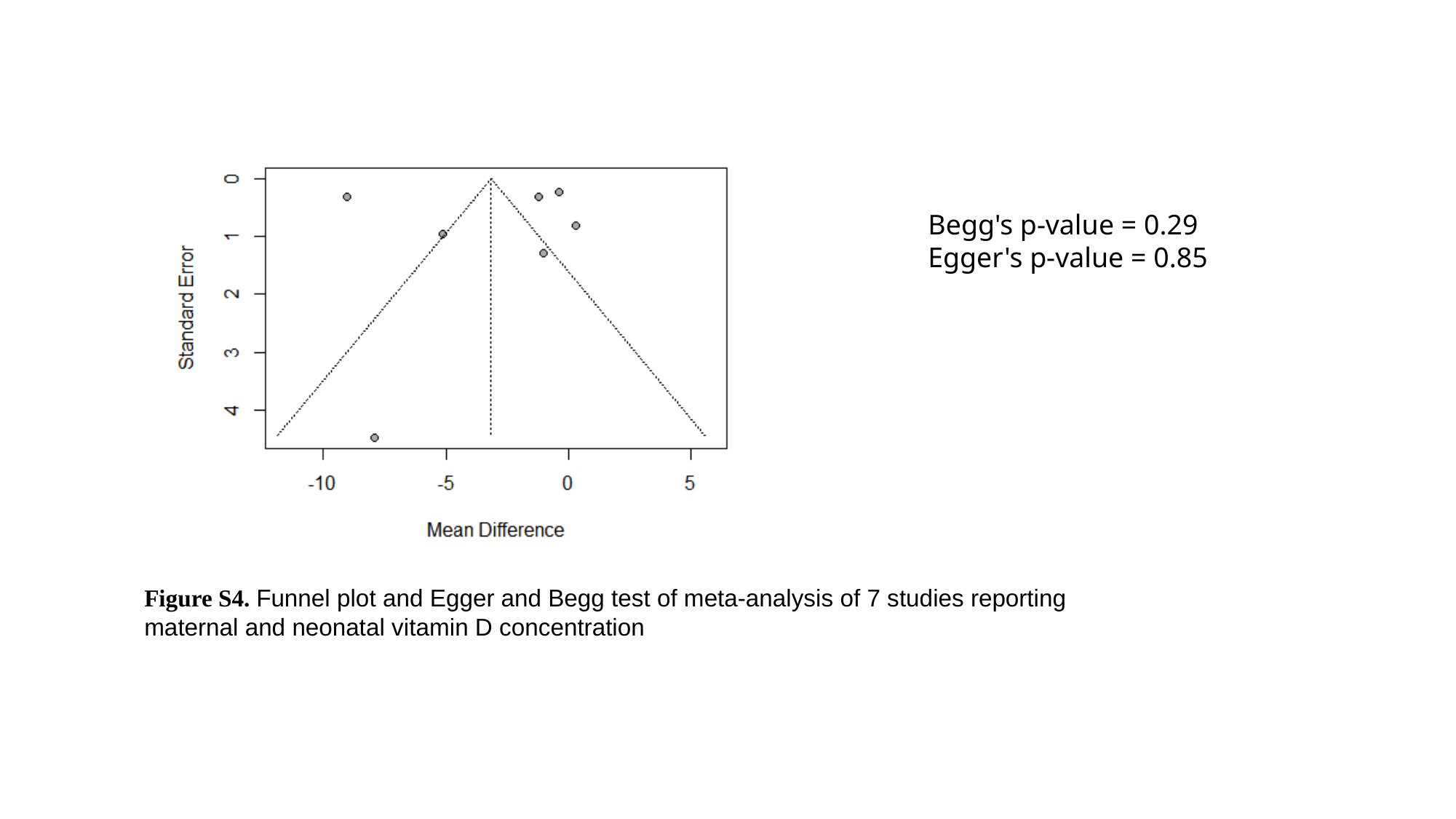

Begg's p-value = 0.29
Egger's p-value = 0.85
Figure S4. Funnel plot and Egger and Begg test of meta-analysis of 7 studies reporting maternal and neonatal vitamin D concentration

## Slide 5
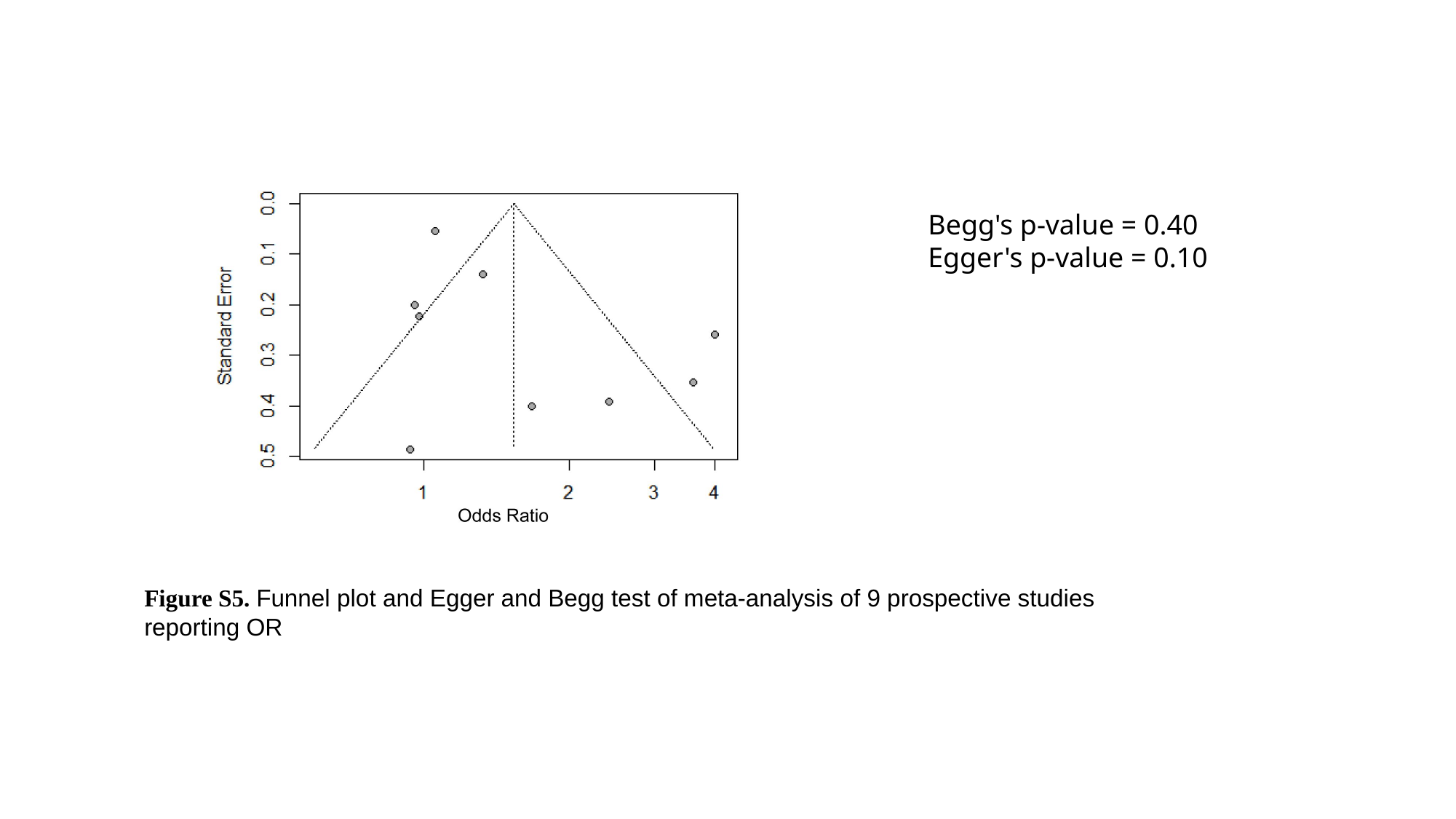

Begg's p-value = 0.40
Egger's p-value = 0.10
Figure S5. Funnel plot and Egger and Begg test of meta-analysis of 9 prospective studies reporting OR
